# Breast cancer survival among young women: a review of the role of modifiable lifestyle factors

**DOI:** 10.1007/s10552-016-0726-5

**Published:** 2016-03-12

**Authors:** Darren R. Brenner, Nigel T. Brockton, Joanne Kotsopoulos, Michelle Cotterchio, Beatrice A. Boucher, Kerry S. Courneya, Julia A. Knight, Ivo A. Olivotto, May Lynn Quan, Christine M. Friedenreich

**Affiliations:** Department of Cancer Epidemiology and Prevention Research, CancerControl Alberta, Alberta Health Services, Room 513, Holy Cross Centre, Box ACB, 2210-2nd St. SW, Calgary, AB T2S 3C3 Canada; Department of Oncology, Cumming School of Medicine, University of Calgary, Calgary, AB Canada; Department of Community Health Sciences, Cumming School of Medicine, University of Calgary, Calgary, AB Canada; Women’s College Research Institute, Women’s College Hospital, Toronto, ON Canada; Dalla Lana School of Public Health, University of Toronto, Toronto, ON Canada; Department of Nutritional Sciences, University of Toronto, Toronto, ON Canada; Prevention and Cancer Control, Cancer Care Ontario, Toronto, ON Canada; Faculty of Physical Education and Recreation, University of Alberta, Edmonton, AB Canada; Lunenfeld-Tanenbaum Research Institute, Mount Sinai Hospital, Toronto, ON Canada; Department of Surgery, Cumming School of Medicine, University of Calgary, Calgary, AB Canada

**Keywords:** Breast cancer, Young onset, Epidemiology, Lifestyle, Modifiable factors, Survival

## Abstract

Almost 7 % of breast cancers are diagnosed among women age 40 years and younger in Western populations. Clinical outcomes among young women are worse. Early age-of-onset increases the risk of contralateral breast cancer, local and distant recurrence, and subsequent mortality. Breast cancers in young women (BCYW) are more likely to present with triple-negative (TNBC), TP53-positive, and HER-2 over-expressing tumors than among older women. However, despite these known differences in breast cancer outcomes and tumor subtypes, there is limited understanding of the basic biology, epidemiology, and optimal therapeutic strategies for BCYW. Several modifiable lifestyle factors associated with reduced risk of developing breast cancer have also been implicated in improved prognosis among breast cancer survivors of all ages. Given the treatment-related toxicities and the extended window for late effects, long-term lifestyle modifications potentially offer significant benefits to BCYW. In this review, we propose a model identifying three main areas of lifestyle factors (energy imbalance, inflammation, and dietary nutrient adequacy) that may influence survival in BCYW. In addition, we provide a summary of mechanisms of action and a synthesis of previous research on each of these topics.

## Introduction

 Breast cancer is the most common cancer among women in Western populations with a lifetime cumulative incidence probability of one in nine [1]. Approximately 6.6 % of breast cancers are diagnosed among women age 40 and younger. The average risk of developing breast cancer by age 40 is one in 173 [1, 2]. Of all cancers diagnosed among women by age 40 years, 40 % are breast cancers. Traditionally, breast cancers in young women (BCYW) have been thought to be etiologically driven primarily by genetic/hereditary factors [3]. BCYW are more likely to be associated with increased familial risk, but only a relatively small proportion of cases (<10 %) are attributable to inherited germline variations in the known familial breast cancer risk genes (*BRCA1/BRCA2*) [4, 5] and are highest in those women with very strong family histories of breast or ovarian cancer [5]. Other genomic factors, including mutations in tumor suppressor and oncogenes, copy number variation, and epigenetics, are likely implicated in cancer initiation and progression among young women. However, these alterations do not fully explain carcinogenesis and subsequent progression among young women.

There are four clinically relevant breast cancer phenotypes currently recognized [6]: luminal A (ER+, PR+, HER2−, Ki67 low), luminal B (ER+, HER2−, PR−, or Ki67 high), triple-negative breast cancer (TNBC; ER−, PR−, and HER2−), and HER2 over-expressing tumors (HER2+). The TNBC and HER2+ subtypes are the most aggressive forms of breast cancer and are over-represented in BCYW [7–9]. Approximately 26 % of BCYW are TNBC compared to 12 % overall [10, 11]. Next-generation sequencing of TNBC has suggested that actionable mutations occur in only a small subset (<20 %) of these cancers [12] and do not completely predict survival [13]. Therefore, non-genomic factors, including lifestyle and other epidemiologic factors, may significantly impact recurrence and survival in BCYW.

**Fig. 1:**
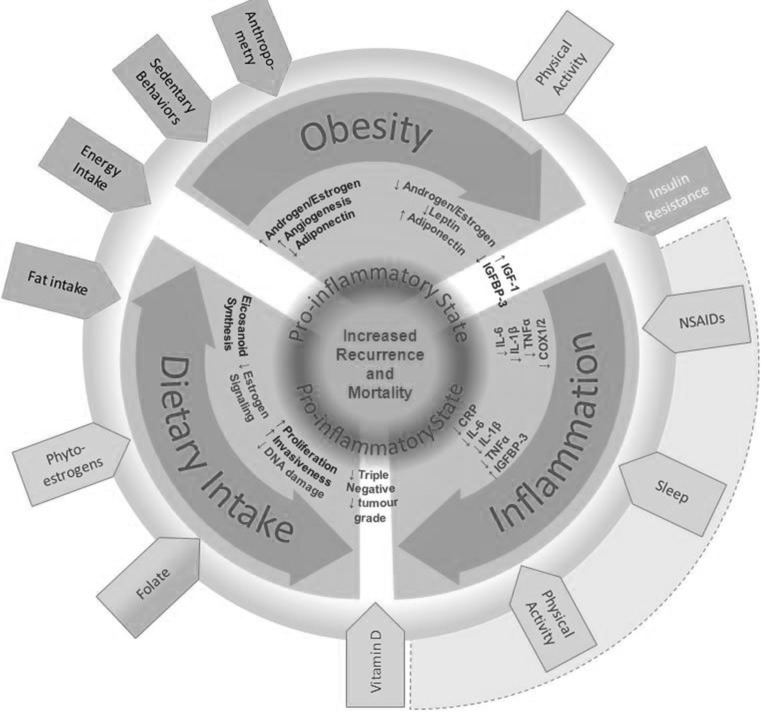
Proposed biologic model depicting how multiple lifestyle risk factors may influence breast cancer prognosis through a pro-inflammatory state including important exposures, pathways, and impact on the candidate biologic mediators *Green arrows* indicate potentially beneficial factors; *red arrows* indicate potentially detrimental factors. (Color figure online)

Here we present a review of the epidemiologic literature on the associations between lifestyle factors, recurrence, and survival for BCYW, defined as a breast cancer diagnosed by age 40 years. The aim of this review is to provide an overview of the associations observed to date among BCYW and how they compare to those that have been observed in the general population of breast cancer survivors. In doing so, we have also aimed to identify gaps in the literature where additional research is needed in this population. BCYW is considered to be distinct from pre-menopausal breast cancer (average age at menopause among North American women is 51 years) [14, 15]. However, since there is limited epidemiologic research specifically examining the effect of candidate risk factors on BCYW, we have included studies on pre-menopausal cases if no BCYW-specific data were available. We propose a biologic model for the impact of selected lifestyle factors on prognosis in BCYW (Fig. 1). In doing so, we provide a conceptual model for the inter-play between these factors and their possible role in cancer progression. We have focused our review on modifiable factors for which, despite evidence for an impact among the general population of breast cancer patients, the research specifically addressing BCYW is limited and needs further investigation.

## A conceptual model for lifestyle factors in breast cancer prognosis among young women

**Fig. 2:**
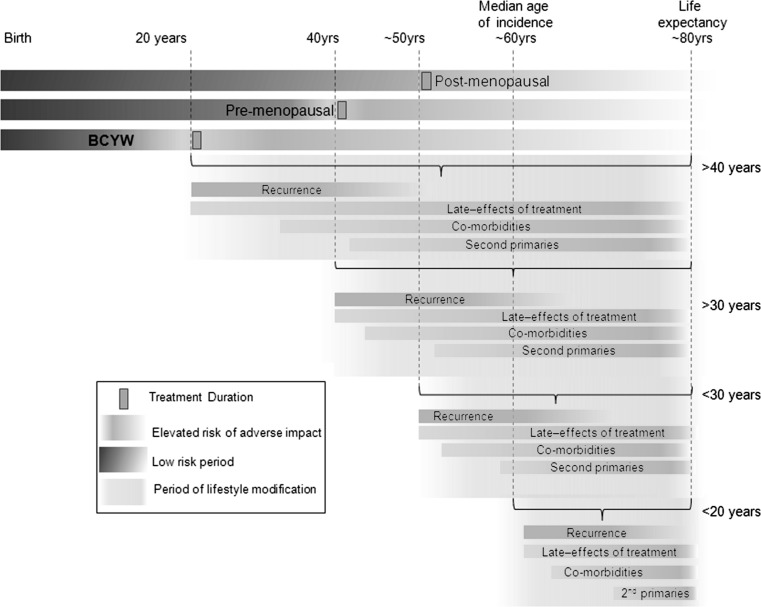
Potential period for lifestyle modification to impact outcomes in breast cancer patients according to age at first diagnosis Compared to women at the median age of diagnosis, young women (<40 years) have approximately twice the duration of post-diagnostic/post-treatment life during which their risk of disease and treatment-related consequences are elevated. The opportunity to mitigate these effects through lifestyle modification is much greater for younger women

Despite the acknowledged differences in clinical outcomes, tumor subtypes and treatment approaches in young women compared with older women, specific insights into the basic biology, epidemiology, and optimal therapeutic strategies for BCYW are relatively sparse. Several modifiable factors that have been associated with reduced risk of developing breast cancer may also be implicated in improved prognosis. Given the treatment-related toxicities, and the extended window for late effects, including cardiotoxic effects of systemic and radiation therapy [16–19], bone health issues [20], and elevated risk of second primary cancers [21, 22], these long-term lifestyle factors potentially offer particularly significant benefits in BCYW (Fig. 2). We propose a biologic model to identify three main lifestyle-related factors in BCYW prognosis research (Fig. 1). We hypothesize that positive energy balance (obesity and physical inactivity), specific dietary factors, and inflammatory triggers contribute to a pro-inflammatory state that is conducive to increased risk of progression, recurrence, and decreased survival after a breast cancer diagnosis in young women. The extended period of potential post-diagnostic survival and the lower burden of competing mortality risks in young women provide a context in which lifestyle modification could have a substantial impact on long-term mortality and morbidity.

All of the factors included in our review affect the hypothesized pro-inflammatory state, and our conceptual model seeks to integrate the impact of lifestyle factors that have been implicated in modifying clinical outcomes following a diagnosis of breast cancer (Fig. 1). The contributors to a pro-inflammatory state (*red labels*) and the potentially beneficial impacts (*green labels*) of diet, physical activity, sleep, and non-steroidal anti-inflammatory drugs (NSAIDs) are included. The conceptual model provides a framework for this review as well as a roadmap for future research to integrate diet, nutrient levels, shared signaling between obesity and inflammation, and potential lifestyle interventions. Each of the three main areas is reviewed in the sections below.

## Clinical context

The cumulative incidence of breast cancer rises exponentially until age 40 years and then rises more linearly with age [23]. Incidence rates among young women vary by geographic region, and ethnicity with the highest rates reported among Western populations [24, 25] and among black women [26]. Clinical outcomes of BCYW are relatively poor compared to older women diagnosed with breast cancer. Early age-of-onset increases the risk of contralateral breast cancer [27], local [28–30], and distant recurrence [23]. The European Organisation for Research and Treatment of Cancer (EORTC) trials showed a hazard ratio (HR) of 2.8 (95 % confidence interval [CI] 1.4–5.6) for local recurrence in patients <35 years compared to those >50 [28]. Voogd et al. [31] examined two large clinical trials of women with stage I–II breast cancer and reported a dramatically increased risk (HR = 9.2, 95 % CI 3.7–23.0) of local recurrence in women <35 years of age compared to women age ≥65 years.

Young age at diagnosis is associated with reduced survival, even after controlling for differences in the distribution of prognostic features between older and younger women with breast cancer [9, 32–35]. A retrospective evaluation of outcomes among more than 200,000 women with breast cancer in the SEER database found that women aged <40 were 39 % more likely to die of their disease when compared to those aged ≥40 (HR = 1.39, 95 % CI 1.34–1.45). The largest differences in mortality were seen among women with early-stage disease. Women <40 were more likely to die of stage I or II cancers than older women (44 and 9 %, respectively) [36]. Similar associations between reduced survival in younger, early-stage patients have been observed in other cohorts of BCYW [37, 38]. Furthermore, survival rates were comparatively lower across all histologic subtypes and stages for women <40 years of age [39]. According to SEER data, for cases diagnosed between 1975 and 2000, the 5-year relative survival rate was 84–86 % overall among women aged 45–80 years, but 80, 76, 72, and 75 % among women aged 35–39, 30–34, 25–29, and 20–25 years, respectively [40].

The absolute benefits of treatment, whether local or systemic, tend to be larger in younger women because of the higher proportion of aggressive disease [41, 42], but few trials have specifically evaluated treatment selection for younger women; the median age of patients in most randomized controlled trials (RCTs) is about 55 years [41–45]. Younger patients tend to have fewer comorbidities and better tolerance of treatment toxicities and, consequently, are often treated more aggressively. Most women are treated with breast-conserving surgery (BCS) followed by radiation therapy (RT) [41, 44, 46–49], but population-based analyses have shown higher rates of mastectomy for BCYW compared to older women with similar stage disease [46, 47]. Residual concerns regarding higher overall recurrence rates, a longer potential life span requiring ongoing follow-up/surveillance, and the risk of radiation-induced second primary tumors in young women [50] may contribute to provider recommendations or patient preference for mastectomy in spite of evidence that outcomes following BCS + RT of mastectomy are equivalent, even among BCYW [31, 51–54]. The 2015 National Comprehensive Cancer Network (NCCN) breast cancer management guidelines no longer cite young age (<35 years) as an indication for mastectomy, as was the case in the earlier 1997 NCCN guidelines [55]. However, young women (<40 years) were almost four times more likely to receive bilateral mastectomy than women aged 50–64 years (OR 3.81; 95 % CIs 3.55–4.08) [56].

Younger women also tend to receive more intensive radiation and systemic therapy despite an international expert panel advocating that young age alone should not be sufficient justification for aggressive therapy [15]. Whole breast RT plus a supplementary “boost” dose to the primary site is routinely administered following BCS in younger women [57] because RCTs have shown that they experience the largest absolute improvements in local control [58]. The majority of young women with breast cancer are candidates for chemotherapy and anti-estrogen therapy (for ER-positive disease, usually with tamoxifen) for at least 5 years [3, 15, 42]. Young women increasingly receive neoadjuvant chemotherapy prior to definitive local therapy to downstage disease and, in some cases, to facilitate BCS where mastectomy might have otherwise been indicated [57]. In addition, the tumor subtypes most commonly found in young women are more likely to respond to neoadjuvant chemotherapy and provide an in vivo evaluation of response to systemic therapy. The higher frequency of HER2 over-expression, TNBC, and *BRCA* mutations in BCYW results in these women being offered novel combinations of systemic therapeutic agents more frequently than older women, particularly within clinical trials.

## Lifestyle factors

### Positive energy balance

#### Obesity

Positive energy balance, as a consequence of excess caloric intake and/or insufficient energy expenditure, results in increased adipose tissue leading to overweight and/or obesity. Both obesity [59] and physical inactivity [60] increase the risk of breast cancer development in older women and progression at all ages. Increased risks for overall mortality and breast cancer-specific mortality associated with increasing body mass index (BMI; e.g., HR = 1.41, 95 % CI 1.29–1.53) [61] or waist–hip ratio (HR = 1.31, 95 % CI 1.08–1.58) were reported in a recent meta-analysis [62]. Larger effect sizes for breast cancer mortality are associated with obesity among pre-menopausal (HR = 1.75, 95 % CI 1.26–2.41) compared to post-menopausal women (HR = 1.34, 95 % CI 1.18–1.53) [61].

One of the biologic mechanisms through which obesity could affect cancer survival is by altering the insulin resistance (IR) pathway [63]. Homeostatic model assessment (HOMA) is a method for assessing β-cell function and IR from basal (fasting) glucose and insulin or C-peptide concentrations [64]. Although no data exist specifically for young women, increasing HOMA-IR has been associated with a positive trend for breast cancer recurrence in ER-/PR-negative patients (*p* for trend = 0.087) and an inverse trend in ER-/PR-positive patients (*p* for trend = 0.081) among a general population of breast cancer patients [65]. In a multiethnic cohort of 527 women with breast cancer [Health, Eating, Activity, and Lifestyle (HEAL) Study], increasing HOMA scores were associated with reduced breast cancer survival (HR = 1.12, 95 % CI 1.05–1.20) [66].

Excess adiposity may also impact cancer survival by altering levels of circulating adipokines, particularly elevating leptin, and decreasing adiponectin [67, 68]. Leptin and adiponectin are secreted by adipose tissue and play opposing endocrine, paracrine, and autocrine roles in the development and progression of breast cancer [69–72]. High (>median) levels of adiponectin were associated with significantly decreased mortality in the HEAL study (HR = 0.39, 95 % CI 0.16–0.95) [66]. Adiponectin induces apoptosis in dose-dependent manner and interacts with estrogen receptors to reduce breast cancer cell growth [73–77]. To our knowledge, the effects of these pathways (insulin resistance and adipokines) have not been evaluated specifically among BCYW; however, differences in survival associated with BMI may be mediated by these pathways in BCYW.

#### Physical activity

Physical activity has been consistently associated with improved survival and other breast cancer-specific outcomes in breast cancer patients [63, 78]. While these epidemiologic studies have included women under 40 years of age, only one study focused specifically on young women (*n* = 717) with 251 breast cancer deaths after a median follow-up of 10.4 years [79]. The risk of breast cancer-specific mortality for young active women (aged 20–54, ≥5 h of recreational activity per week) was reduced compared to young inactive women (HR = 0.78 (95 % CI 0.45–1.34)) [80]. This estimate was adjusted for cancer stage and BMI, but not for treatment. A recent meta-analysis reported pooled data from four prospective cohort studies of older/post-menopausal women [81]; those engaging in at least 10 MET-hours (MET = metabolic equivalent) of physical activity per week had a 27 % reduction in all-cause mortality (*n* = 1,468 events, HR = 0.73, 95 % CI 0.66–0.82) and a 25 % reduction in breast cancer-specific mortality (*n* = 971 events, HR = 0.75, 95 % CI 0.65–0.85) compared with women performing <10 MET-hours/week [81]. The magnitude of effect in BCYW is unknown. No studies have examined the role of sedentary behavior (commonly conceptualized as sitting time) in breast cancer survival, despite its emergence as a distinct risk factor for breast cancer separate from the beneficial effects of physical activity [82].

In the only randomized controlled trial to date, Courneya et al. [83] reported an exploratory follow-up of breast cancer outcomes in 242 breast cancer patients randomized to supervised exercise (aerobic or resistance) or usual care during adjuvant chemotherapy. After an 8-year follow-up, the overall risk of a disease-free survival event for the exercise groups was reduced, although the difference was not significant [HR = 0.68 (95 % CI 0.37–1.24)]. This risk reduction was slightly lower for women under 50 years of age (HR = 0.77, 95 % CI 0.32–1.84). Interestingly, there did not appear to be any suggestion of benefit for women with TNBC (HR = 1.25, 95 % CI 0.40–3.95), but there was for women with HER2+ breast cancer, although nonsignificant (HR = 0.21, 95 % CI 0.04–1.02). These data suggest a potentially complex effect of exercise on breast cancer outcomes in BCYW that may vary by breast cancer subtype.

Several biologic mechanisms have been proposed for the beneficial effects of physical activity in cancer progression including changes in BMI and adiposity which are likely to impact the biologic pathways discussed above as well as altered levels of estrogens, androgens, sex hormone-binding globulin, and reduced levels of inflammatory markers [84].

## Inflammation-related factors

Inflammation represents a complex network of biologic responses and pathways mediated by cytokines, lymphocytes, acute-phase proteins, prostaglandins, and many other cellular components. Inflammation plays a pivotal role in the development and progression of breast cancer [85] and is considered a hallmark of cancer [86]. Exposures that increase levels of inflammation and subsequent biomarkers of inflammation are associated with reduced overall survival among breast cancer patients [87]. The subsequent sections review several inflammatory factors and their potential roles in BCYW survival.

### Cytokines

Inflammatory cytokines including interleukins (IL-1β, IL-2, IL-6, IL-8, IL-12), tumor necrosis factor alpha (TNF-α), and interferon gamma (IFN-γ) are key inflammatory mediators of interest in breast cancer progression. Changes in inflammatory cytokine levels are observed in obese individuals and may represent critical mediators between energy imbalance and breast cancer survival [88]. Elevated circulating levels of IL-6 have been associated with reduced breast cancer survival [89] and increased tumor burden [90]. IL-8 is highly expressed by tumor and stromal cells, and its expression in breast tumor cells is stimulated by TNF-α and/or IL-1β, two other important inflammatory cytokines in cancer development [91, 92]. The mean survival time from first metastasis was significantly lower in breast cancer patients with reduced circulating IL-2 concentrations compared to those with normal IL-2 values, irrespective of response to therapy and dominant metastasis sites [93]. Serum levels of TNF-α have been found to be significantly predictive of breast cancer survival, particularly among women with HER-2 over-expression [94, 95].

### T lymphocytes

The immune system may play an important role in tumor control through both the elimination of immunogenic tumor cells (immunosurveillance) and to promote the outgrowth of less immunogenic tumor cell variants (immune editing) [96]. In breast cancer, the relationship between host defense mechanisms and clinical outcomes has been debated for some time [97]. T cells play an integral role in the inflammatory response. For example, T-helper (Th)1 cells produce cytokines IL-2, IFN-ã, and TNF-á, all of which are important in viral clearance and tumor surveillance. In contrast, Th2 cells produce IL-4, IL-5, and IL-13 which help to activate eosinophils and mast cells. Meanwhile, CD8-expressing cytotoxic T lymphocytes (CTLs) secrete cytokines and target the cells with which they interact for destruction [98]. Therefore, both groups of T cells may mediate processes integral to inflammation, carcinogenesis, and cancer progression [99]. An analysis of 1,334 tumors showed that a high total CD8+ CTL count was an independent prognostic factor associated with longer survival in early-stage breast cancer patients (*p* < 0.001) [100]. The presence of a CD8+ lymphocytic infiltrate in breast cancer tissue is associated with improved outcome, further indicating that the immune system participates in the control and elimination of tumor cells [101, 102]. Conversely, high levels of CD4+ CTL infiltration have typically been correlated with reduced overall survival [103]. No studies have directly examined the prognostic impact of T cell levels in BCYW, but T cell infiltrate may be particularly significant in TNBC which are more common in BCYW [99, 104]. The immunomodulatory subtype of TNBC [105] has been characterized by elevated expression of genes involved in T cell function, immune transcription, interferon (IFN) response, and antigen processing [106]. Immunotherapy and lymphocytic response to therapy are of interest in breast cancer treatment [107, 108], but the prognostic effects and potential for immune-based intervention have not yet been investigated in BCYW.

### Acute-phase proteins

Acute-phase proteins are produced by the liver in response to inflammatory cytokines. Elevated levels of serum amyloid A (SAA) and C-reactive protein (CRP) are associated with significantly increased risk of death among breast cancer patients (HR = 3.15, 95 % CI 1.73–5.65 and HR = 2.27, 95 % CI 1.27–4.08, respectively) [109, 110]. However, it is unclear whether these proteins have direct functional roles or simply reflect an overall inflammatory response.

### NSAID use

Prostaglandins are lipid autacoids derived from arachidonic acid, an omega-6 fatty acid. They both sustain homeostatic functions and mediate pathogenic mechanisms, including the inflammatory response [111]. Prostaglandins are synthesized by cyclooxygenases COX-1 and COX-2. Elevated COX-2 expression has been consistently associated with advanced disease stage, reduced survival, and poor prognosis among breast cancer patients [112–114]. The potential anti-metastatic properties of aspirin have been reported for several decades [115, 116], but it is only recently that significant epidemiologic research has focused on the role of aspirin and other NSAIDs in the prognosis of breast cancer patients [117]. Existing literature suggests that the proportion of breast cancer patients regularly using NSAIDs varies from 30 to 50 % (with varying definitions of regular use). Recent data indicate that post-diagnostic NSAID use is associated with reduced likelihood of breast cancer recurrence and breast cancer-specific mortality among the general population of breast cancer patients [118–122]. Although no previous studies have focused specifically on the impact of NSAIDS on survival among BCYW, those studies that examined survival differences across pre- and post-menopausal status have not reported significant differences although statistical power was generally limited. However, Zhang et al. [123] reported that the reduced risk of breast cancer (OR = 0.62; 95 % CI 0.41-0.94) associated with NSAID use was restricted to pre-menopausal women only. The dearth of studies examining the impact of NSAID use on BCYW is likely attributable to both the relative rarity of BYCW and indications for chronic NSAID use among young women.

### Sleep

Sleep disturbance and insomnia are common and often persistent behavioral comorbidities following a diagnosis of cancer [124]. Most sleep and breast cancer research, including circadian disruption, has focused on the impact of sleep quality and quantity on breast cancer risk [125], but there is increasing evidence that these factors may impact both quality of life and survival outcomes after a breast cancer diagnosis [125, 126]. Sleep affects many of the inflammatory factors that are implicated in our proposed biologic model (Fig. 1) such as cytokine production, adipokine production, and immune responses [127, 128]. Furthermore, sleep disturbance, insomnia, and sleep restriction are associated with pro-inflammatory responses [129] and these responses are particularly pronounced in women. The methodological challenges of examining the inflammatory and immune consequences of compromised sleep are substantial [128]. However, chronic sleep curtailment has been associated with elevated inflammatory activity [130, 131] although no studies have specifically investigated these responses in young women with breast cancer.

## Dietary intake

There is considerable evidence regarding the role of dietary intake in breast cancer etiology, but relatively little research has focused on the role of these exposures in prognosis, particularly among BCYW. The World Cancer Research Fund states that “…in the absence of stronger evidence, we believe the best advice for cancer survivors is to follow our Recommendations for Cancer Prevention” [105]. There is some evidence to suggest that high post-diagnostic fruit, vegetable, whole grain, and protein intake decrease the risk of mortality following breast cancer, while high animal fat intake increases the risk [132, 133]. The role of specific dietary components, including vitamins, fatty acids, and alcohol consumption, or overall dietary patterns, have also been evaluated, but findings are inconclusive [133–137]. Well-designed studies are needed to address the critical gap in the current literature regarding the role of diet in breast cancer survival, particularly among young women. In the subsequent sections, we review several dietary factors that may modify survival among young women with breast cancer. Each factor has been associated with altered breast cancer risk or survival in breast cancer cohorts not restricted by age. Where relevant, we relate these dietary factors to our proposed biologic model (Fig. 1).

### Dietary fat

Dietary fat intake has been extensively researched in relation to breast cancer risk, but the evidence remains inconclusive [138]. Dietary fat intake might influence risk of breast cancer through the promotion of oxidative stress, hormonal dysregulation, or inflammatory signaling [139]. These same mechanisms are implicated in breast cancer progression and recurrence, but few studies have investigated the impact of dietary fat intake on breast cancer outcomes and none have specifically investigated their effects on BCYW.

The literature on dietary fat and breast cancer survival has recently been thoroughly reviewed elsewhere [139]. Several epidemiologic studies have analyzed the association between pre-diagnostic and post-diagnostic fat intake on survival in breast cancer patients, with total dietary fat intake the most common measure. Fewer studies have considered the contribution of subtypes of fat (e.g., transfat and saturated fat) in breast cancer patients although these studies have reported the strongest associations with mortality [140]. More studies have investigated overall survival than breast cancer-specific survival, and the reported associations with overall survival are typically stronger than that for breast cancer-specific mortality. In keeping with our biologic model, the caloric density and pro-inflammatory effects of dietary fat could adversely affect outcomes in young women with breast cancer through multiple biologic processes (Fig. 1). Many of the studies investigating the role of dietary fat intake on breast cancer outcomes have reported associations that fail to reach statistical significance, and the point estimates are too inconsistent to provide a clear consensus [139]. However, the lack of published research regarding the role of fat intake in BCYW combined with both the potential beneficial and detrimental impacts of fat subtypes, presents an opportunity to address the impact of fat intake on post-diagnostic outcomes in BCYW.

### Vitamin D

Vitamin D is mostly synthesized in the skin by ultraviolet B radiation (only in the summer at higher latitudes), while dietary intake and supplements also contribute to overall vitamin D status, particularly in the winter in northern populations. The evidence to support an inverse association between breast cancer risk and vitamin D status is complex and uncertain [141], but there is increasing evidence that vitamin D status may be an important factor in breast cancer survival [142]. There are several plausible mechanisms whereby vitamin D may influence the phenotype of the primary tumor and potentially improve outcomes. Vitamin D exhibits pro-differentiation and anti-proliferative properties [143]. Accumulating evidence implicates suboptimal vitamin D status in the development of inflammatory and immunological conditions [144], compatible with the observed immunosuppressive and anti-inflammatory activity of 1,25-dihydroxyvitamin D, the active metabolite of vitamin D [145]. No previous studies have examined the impact of vitamin D status in BCYW, and the impact on pre- versus post-menopausal women has been inconsistent. However, a few studies have identified differences in outcome between tumor phenotypes in pre- and post-menopausal women associated with vitamin D status. Goodwin et al. [142] reported that low serum 25-hydroxyvitamin D concentration (<50 nmol/L) was associated with higher tumor grade at diagnosis, increased risk of distant recurrence (HR = 1.94, 95 % CI 1.16–3.25), and increased risk of breast cancer-specific death (HR = 1.73, 95 % CI 1.05–2.86) compared with patients with levels >72 nmol/L. Similarly, Yao et al. [146] reported that low vitamin D status was associated with the occurrence of higher-grade tumors in pre-menopausal women only. Peppone et al. [147] observed an increased proportion of ER-negative tumors in the vitamin D-deficient group (<20 ng/mL) compared to patients with higher levels. Not all studies have suggested higher serum vitamin D post-diagnosis is associated with better outcomes [148, 149]. These conflicting results are partly attributable to insufficient statistical power, non-population-based sampling, and suboptimal exposure assessment.

### Phytoestrogens

The two main classes of phytoestrogens (plant compounds with non-steroidal estrogen-like structures and activities) are isoflavones and lignans. Isoflavones are found primarily in soy foods, while lignans are found in low concentrations in fiber-rich foods such as grains, legumes, seeds, fruits/vegetables, and in high concentration in flaxseeds [150]. Many supplements also contain high concentrations of isoflavones and lignans [151]. For over a decade, researchers have reported that dietary phytoestrogen intake is associated with reduced breast cancer risk, possibly due to demonstrated beneficial effects on proliferation, apoptosis, angiogenesis, as well as estrogen receptor mediated and other activities, including a range of anti-inflammatory effects [152–158]. However, only recently has the association between post-diagnostic phytoestrogen intake with breast cancer recurrence and mortality been investigated. Overall, most studies suggested that post-diagnosis intake improved prognosis [159–164], but findings stratified by menopausal status, if reported, have been inconsistent [159, 160, 162]. Most of the six prognostic studies included predominately post-menopausal women [159, 161, 163, 164], leaving a paucity of information regarding phytoestrogen intake and prognosis among BCYM. The largest soy/isoflavone study found a significantly reduced risk of recurrence and breast cancer mortality combined (HR = 0.77, 95 % CI 0.60–0.98), with no difference by menopausal status. Two meta-analyses reported a statistically significantly reduced risk of recurrence that was restricted to post-menopausal women [165, 166]. A reduced risk of all-cause and breast cancer-specific mortality was also reported [165, 166]. No prognostic study has assessed post-diagnostic lignan intake, although one study of post-menopausal women evaluated serum levels of enterolactone—a lignan biomarker [164]. Higher serum enterolactone levels were associated with a significantly reduced risk of death (HR = 0.58, 95 % CI 0.34–0.99) and a nonsignificantly reduced risk of recurrence (HR = 0.62, 95 % CI 0.35–1.09). Since prognosis varies by tumor and treatment characteristics, future studies must control for these factors [164, 166].

### Folate

Folate is a naturally occurring, water-soluble vitamin B [167]. Mandatory fortification of food with folic acid, implemented to reduce the incidence of neural tube defects, has resulted in a dramatic increase in folate intake at the population level [168, 169]. Folate plays an important role in DNA synthesis and methylation by mediating the transfer of single-carbon molecules for various biologic reactions [170, 171]. The mechanism by which folate intake or status might impact BCYW and the direction of the association with outcomes is uncertain. Both DNA synthesis and DNA methylation could promote or suppress cancer progression [170, 171]. Given its important biologic role, there has been intense interest in the cancer-protective effects of folate [171, 172]. Although some literature suggests an inverse association between folate status (dietary intake and/or blood levels) and the risk of developing cancer [173], contrasting evidence also supports that high folate levels may promote cancer progression and actually increase the risk of some cancers, including breast cancer [173–175]. High folate intake may increase breast cancer risk by promoting the progression of existing (pre-)neoplastic lesions, by expanding the breast stem cell population or by preventing terminal differentiation in ductal cells [173, 175, 176]. Since a high proportion of cancer survivors consume supplements containing folic acid [177–179], the role of folate in breast cancer prognosis needs clarification.

To date, eight studies have evaluated the relationship between dietary or plasma folate levels and survival after breast cancer and findings have been inconsistent [133, 180–186]. Four studies found no association [133, 181, 182, 185], three reported a significant inverse relationship [180, 183, 184], and a recent study reported a harmful effect of high folate status on breast cancer prognosis, specifically in ER−/PR− tumors [186]. However, these studies included mostly post-menopausal women and none evaluated the impact of folate in patients diagnosed at a young age. It is also unknown whether the relationship between folate status and survival varies by folate receptor alpha (FRα) expression of the tumor. Positivity for FRα has previously been associated with the occurrence of TNBC and poor prognosis [187]. Interestingly, FRβ was recently identified as a marker for a pro-inflammatory subset of monocytes [188]. Both elevated folate and folate insufficiency may induce inflammatory processes [189].

### Alcohol

The role of alcohol consumption in breast cancer development and progression generates great public interest and scientific debate. The exact mechanism of action remains to be elucidated, but alcohol is proposed to cause tissue damage and cancer progression is through the formation of acetaldehyde [190]. Acetaldehyde is the primary product of ethanol oxidation, and its rate of formation is determined by the rate of nicotinamide adenine dinucleotide oxidation through mitochondrial electron transport [191]. Inefficient metabolism or excretion of ethanol and acetaldehyde results in the formation of reactive oxygen species, notably superoxide [192], which affects carcinogenesis through an inflammatory response.

The association between alcohol consumption and prognosis has not been directly evaluated in BCYW. However, a recent meta-analysis of prospective cohort data showed that for pre-menopausal women, high levels of alcohol consumption were associated with increased risk of breast cancer recurrence (HR = 1.52, 95 % CI 1.21–1.90) [136]. Another meta-analysis reported a small reduction in all-cause mortality associated with moderate consumption in ER+ patients and a reduction in breast cancer-specific mortality with moderate consumption in ER-negative breast cancer [193]. The associations overall and across relevant subgroups require additional study because of the high prevalence of the exposure among young women in western populations (54.6 % among women 18–44 in the USA) [194].

## Conclusions and strategies for additional research

Given the high rates of recurrence and poorer survival in BCYW, there is an urgent need to optimize lifestyle advice to improve outcomes for these women. Modifiable lifestyle factors offer an opportunity to complement conventional therapies provided to these women. The majority of the factors included in this review show common etiologic links in the progression of breast cancer through altered pathways ultimately leading to a pro-inflammatory state. We acknowledge that the list of factors is not exhaustive; however, the aim of this review is to provide an integrated view of the inter-relationship between the most promising candidate modifiable factors. In doing so, we provide a conceptual model for the inter-play between factors and their possible contributions to cancer progression and survival. This literature review has highlighted the paucity of data on the effect of these modifiable factors on breast cancer prognosis among young women. We propose that the potential for an impact on reducing recurrence and improving survival may be considerable. Additional research on this area will provide directions for lifestyle and clinical interventions that support beneficial behavioral change to improve outcomes in young women diagnosed with breast cancer. Future investigations should aim to maximize the integration of factors so that the relative impacts of multiple lifestyle factors can be assessed both independently and in combination. Furthermore, molecular and genetic markers may help to determine potentially relevant subgroups likely to experience maximal or minimal beneficial impact. Integrated study designs with appropriate biospecimen collections will support the assessment of the relative contributions of these exposures to the pathways proposed in our biologic model focused on survival in BCYW.
